# The prospective association of prenatal anxiety symptoms in mothers and fathers with general child development 14 months postpartum and the mediating role of parent-child bonding: a mediation analysis within the longitudinal cohort study DREAM

**DOI:** 10.1186/s12884-025-07846-z

**Published:** 2025-09-09

**Authors:** Anna C. von Olberg, Victoria Weise, Judith T. Mack, Kerstin Weidner, Susan Garthus-Niegel

**Affiliations:** 1 Institute and Policlinic of Occupational and Social Medicine, Faculty of Medicine, Technische Universität Dresden, Fetscherstraße 74, Dresden, 01307 Germany; 2 Department for Psychotherapy and Psychosomatic Medicine, Carl Gustav Carus Faculty of Medicine, Technische Universität Dresden, Dresden, Germany; 3 Institute for Systems Medicine (ISM), Faculty of Medicine, Medical School Hamburg, Hamburg, Germany; 4 Department of Childhood and Families, Norwegian Institute of Public Health, Oslo, Norway

**Keywords:** DREAM study, Anxiety, Child development, Mother–child bonding, Father-child bonding, Depression, Perinatal, Mediation analysis

## Abstract

**Background:**

Anxiety symptoms during pregnancy are a frequent mental health issue for expectant mothers and fathers. Research revealed that prenatal anxiety symptoms can impact parent-child bonding and child development. This study aims to investigate the prospective relationship between prenatal anxiety symptoms and general child development and whether it is mediated by parent-child bonding. Considering the paucity of perinatal research on fathers, their inclusion is of particular interest.

**Methods:**

Data were derived from the prospective cohort study DREAM including 1,544 mothers and 985 fathers. Anxiety symptoms were assessed during pregnancy; parent-child bonding eight weeks after childbirth; and general child development 14 months postpartum via questionnaires. Mediation analyses were conducted. It was controlled for several perinatal confounding factors in a second model. Postnatal depression symptoms were added to the model as confounding factor in a third step to study its influence separately.

**Results:**

In this population-based sample, prenatal anxiety symptoms were more pronounced in mothers than in fathers, whereas the quality of parent-child bonding was very similar for both parents. No significant association was found between prenatal anxiety symptoms and general child development. But prenatal anxiety symptoms predicted poorer parent-child bonding, also when controlling for confounders (mothers: β =.154; *p* <.001; fathers: β =.152; *p* = <.001). However, this effect disappeared when postnatal depression symptoms were additionally controlled for. In turn, parent-child bonding predicted impaired general child development, even when controlling for all confounders (mothers: β = -.104; *p* =.002; fathers: β = -.104; *p* =.012). Accordingly, the indirect effect was significant (mothers: β = -.002; *BCa 95%CI* = [-0.137;0.053]; fathers: β = -.004; *BCa 95%CI* = [-0.354;0.098]) and therefore parent-child bonding mediated the association between prenatal anxiety symptoms and general child development. However, only when postnatal depression symptoms were not controlled for. These associations did not differ between mothers and fathers.

**Conclusion:**

Parent-child bonding is relevant for child development, especially in the presence of prenatal anxiety symptoms. This is the case for both parents, therefore fathers should be included more frequently in perinatal research and clinical practice as their mental health and bonding appear to be equally important. Furthermore, it is important to address parent-child bonding in clinical care, especially when mothers or fathers suffer from anxiety or depression symptoms.

**Supplementary Information:**

The online version contains supplementary material available at 10.1186/s12884-025-07846-z.

## Background

### Prenatal anxiety

The birth of a child is a life-changing event that poses various challenges for mothers and fathers [1]. In the perinatal period, the risk of developing a mental health disorder is increased [2, 3], with anxiety disorders representing the most common mental health disorders in the perinatal population [4–7] affecting approximately 22% of mothers and 10% of fathers [8]. Anxiety symptoms are characterized by physiological reactions such as increased heart rate and sweating, as well as psychological manifestations such as unease, worrying, and restlessness [9].

When these symptoms occur in expectant mothers, they can have serious consequences, including increased risk of hypertensive disorders during pregnancy or preterm birth, lower infant birth weight, reduced breastfeeding rates, postnatal depression symptoms, and greater risk of suicide in mothers [10–13]. In fathers, associations with impaired parenting skills, affected social relationships, and postnatal depression symptoms have been reported [14]. Given the high prevalence and severity of possible consequences, anxiety symptoms experienced during pregnancy constitute a significant public health concern for parents [15, 16], with serious consequences also affecting the child [15].

### Association between prenatal anxiety symptoms and child development

The intrauterine period is a highly sensitive phase of child development, during which adverse influences can have substantial negative impacts [17, 18], also on long-term child development [19, 20]. Beginning at the moment of conception the process of functional maturation of motor, socioemotional, language, and cognitive skills is shaped by a multitude of interacting genetic, biological, and psychosocial factors [21, 22]. There is strong evidence, on the basis of several systematic reviews and meta analyses, that maternal prenatal anxiety symptoms are associated with impaired psychomotor development, cognitive development, child emotional problems, and neurodevelopment [23–27]. Only a few studies have reported divergent findings. These studies indicated small beneficial effects, but only in cases of mild anxiety symptoms [28, 29].

With respect to the consequences of paternal prenatal anxiety symptoms, the body of related research is much smaller. For general paternal perinatal mental health issues, some evidence suggests a negative impact on children’s emotional and behavioral development [30]. Bekkhus et al. [31] reported a very small significant effect between more paternal prenatal anxiety symptoms and child behavior problems, at 1.5 years and 5 years. Another large population-based cohort study also revealed a significant association between paternal prenatal psychological distress and children’s behavioral problems at 3 years [32]. However, they did not differentiate between anxiety and depression; therefore, the isolated effect of anxiety symptoms remains unclear [32]. In contrast, Rogers et al. [33] did not find a significant association between paternal prenatal anxiety symptoms and socioemotional and language development. Hence, the current body of research does not allow any conclusions on the prenatal paternal influence in this specific association.

A possible mechanism for the association between maternal prenatal anxiety symptoms and impaired child development is fetal programming. In the uterus, where vital steps of organ and functional development occur, the effects of maternal stress may be transmitted through the placenta, via hormones, placental blood flow, oxygen saturation, metabolic parameters, and nutritional components, potentially resulting in enduring changes in fetal metabolic, physiological, and structural body functions [26, 34, 35], such as dysregulation of the hypothalamic-pituitary-adrenocortical axis (HPA axis) or alterations in brain development [23, 36, 37]. Moreover, certain dispositions for altered development could be transferred genetically [38].

### Parent-child bonding in this context

However, not only prenatal factors but also the quality of the postnatal relationship and interaction with the primary caregiver, typically the parents, are important for child development [39, 40]. In the relationship between a child and parents, two different aspects are relevant. One is the child’s emotional attachment to the parents [41], and the other is the parents’ emotional, behavioral, cognitive, and neurobiological connection to the child, known as parent-child bonding [41–43]. It is characterized as an enduring, committed, and engaged parental-driven process that begins during pregnancy and continues throughout childhood, with the first year of the child’s life being of special importance [41, 43, 44].

Usually, parents successfully develop an emotional bond with their children. In a Portuguese population-based sample only 4.7% of mothers and 2.4% of fathers reported poor or entirely absent bonding [45]. Moreover, the study revealed that the emotional engagement of both mothers and fathers with their newborn child was similar [45]. In the context of perinatal mental health disorders, poor mother-child bonding is more common, with 10–25% of women affected [46, 47]. Additionally, among women with prenatal anxiety symptoms, the risk for bonding impairment is elevated [48]. However, postnatal depression symptoms seem to have an even stronger association with poor mother-child bonding [44, 48]. Nevertheless, most women suffering from anxiety have no issues forming a strong emotional bond with their offspring [49]. One study even reported a positive association between prenatal anxiety symptoms and postnatal bonding [50]. Similarly, in fathers, mental health problems constitute a risk factor for poor father-child bonding [51]. For example, paternal perinatal depression symptoms have been associated with impaired father-child bonding [52]. A recent pilot study revealed that prenatal anxiety symptoms in fathers were a significant, independent predictor of better father-child bonding [53]. However, fathers’ prenatal anxiety symptoms and father-child bonding have gained very little scientific attention.

Poor parent-child bonding has been associated with child development impairment [43, 51]. Some studies have reported significant effects of mother-child bonding on socioemotional development [54, 55]. Fathers’ poor parent-child bonding has been associated with worse executive functioning at the age of two [56]. But generally, the specific association between the parent-child bonding and child development has been underexplored [57]. The closely related concept of parent-child interaction [43] has received increasing attention and has been consistently linked to all domains of child development, for mothers and fathers [39, 58–61].

### The paternal role

Although fathers play a vital role in the family life, they are traditionally underrepresented in perinatal research [62, 63]. This is problematic because they also significantly shape child development, even before childbirth, due to genetic and epigenetic factors, as well as their relationship with the mother and their influence on the socioeconomic environment [59, 64]. Additionally, society’s perception of the fathers’ role has evolved in recent years, leading fathers to be increasingly involved in childcare [65, 66]. This even expands their influence on the child development and underscores the significance of father-child bonding [62]. Therefore, further investigation of the fathers’ role in the perinatal period is important [30, 67].

### Potential mediation

According to the existing literature, parent-child bonding might be a relevant mediating factor between prenatal anxiety symptoms and child development. This mediation effect has been investigated previously in an Australian prospective cohort study, but only for mothers and considering only socioemotional child development [68]. They reported that parent-child bonding was a significant mediator of the associations between prenatal anxiety, depression, and stress and socioemotional child development. However, to the best of our knowledge, no other study has investigated these specific effects for both mothers and fathers or considered general child development rather than focusing only on socioemotional child development so far.

### Aims of the study

With a better understanding of the mechanisms influencing child development, more effective prevention and treatment interventions can be developed. Regarding the role of fathers in the perinatal period, a growing number of studies has been carried out but still more scientific evidence is needed to further clarify their role and influence, compared to mothers in young families [51, 64]. The further evidence-based inclusion of fathers in the perinatal care could unravel untapped potential for child development. Moreover, it is of interest to investigate whether prenatal anxiety symptoms and parent-child bonding have an impact on overall child development, beyond their known influence on socioemotional development. Regarding the association between perinatal affective disorders and parent-child bonding, the review by O’Dea et al. [48] called for more longitudinal studies to further clarify the influence of prenatal affective disorders on postnatal parent-child bonding, and that studies on the influence of perinatal anxiety disorders are still lacking compared to studies on perinatal depression. This population-based study with its prospective design addresses these needs.

Therefore, the present study aims to show that higher levels of prenatal anxiety symptoms predict more impaired general child development in both mothers and fathers, and that this association is mediated by poorer parent-child bonding. Furthermore, whether these associations differ between mothers and fathers is being explored.

## Methods

### Design

This study is based on data from the cohort study Dresden Study on Parenting, Work, and Mental Health (“DResdner Studie zu Elternschaft, Arbeit und Mentaler Gesundheit”; DREAM). The DREAM study examines the prospective associations between parental work participation, role distribution, perinatal factors, stress factors, long-term family (mental) health, and intrafamily relationships. The study currently consists of seven measurement points: T1 during pregnancy, T2 8 weeks after the anticipated birth date, T3 14 months, T4 2 years, T5 3 years, T6 4.5 years, and T7 7.5 years after birth. The participants complete quantitative questionnaires at each measurement point, either in paper and pencil format or online. In the present study, data from the measurement points T1, T2, and T3 were included. Prenatal anxiety symptoms were assessed during pregnancy (T1), parent-child bonding was assessed at 8 weeks postpartum (T2), and child development was assessed at 14 months of age (T3). This early point in time for measuring child development was chosen because the number of factors influencing child development increases over time, making it more difficult to distinguish and measure the specific effects of prenatal anxiety symptoms later in life. No changes were made to the study design during the data collection period from T1 to T3. More information about the design and methods of the DREAM study can be found in the study protocol [69].

### Sample

The sample is population-based and consists of a total sample size of 3,861 parents, including 2,227 mothers, 1,618 male partners, and 16 female partners. They were recruited from June 2017 to the end of 2020 by team members who personally approached women and their partners attending birth information events in obstetric clinics. Recruitment also took place in midwife practices and through flyers. The inclusion criteria comprise a current pregnancy or being in a long-term relationship with the pregnant woman, being a resident in Dresden, Germany, or the surrounding area, and having sufficient German skills. Participation is entirely voluntary and without financial compensation. However, small incentives (e.g., rompers or books) are gifted with every follow-up questionnaire. All participants provided written informed consent. The current study is based on version 9 of the quality-assured data files. At the time of data extraction (January 31, 2022), prospective data collection was complete for T1 and T2 and ongoing for T3.

**Fig. 1:**
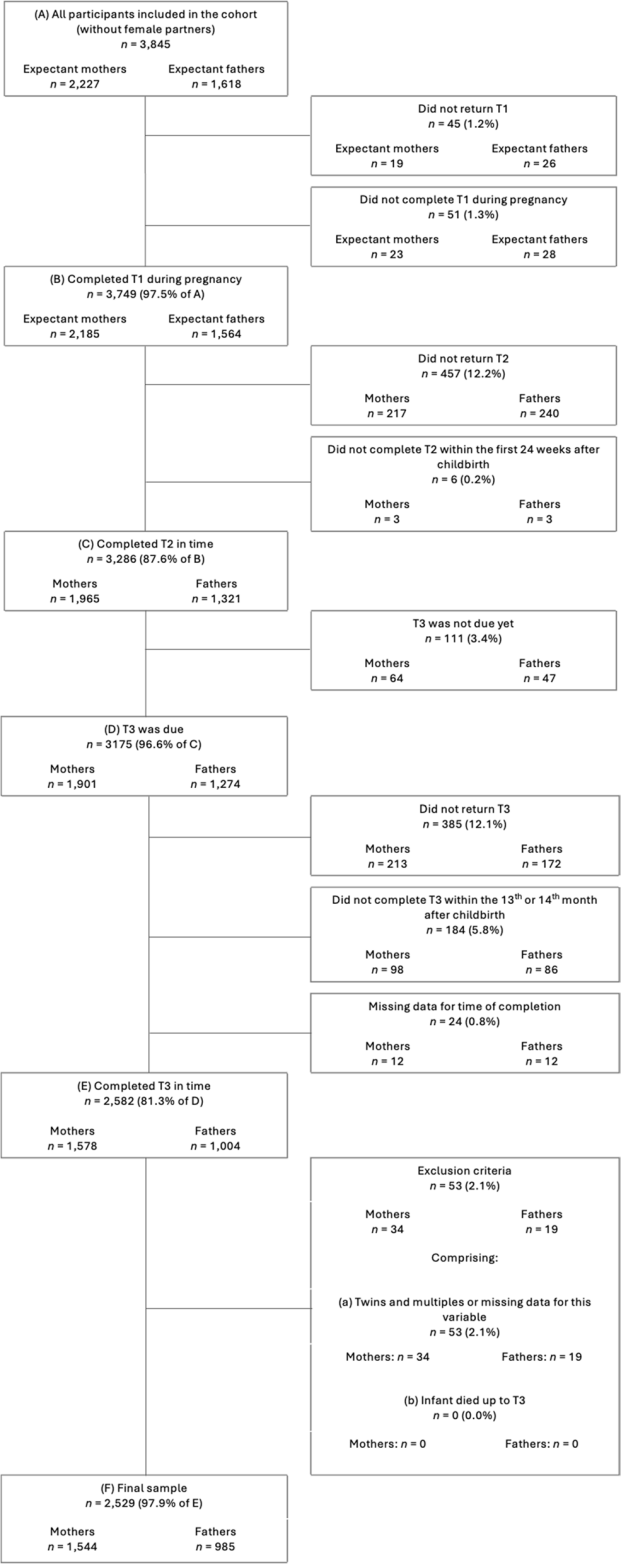
Flowchart of retention rate and exclusion criteria resulting in final sample T1 = during pregnancy; T2 = 8 weeks after childbirth; T3 = 14 months childbirth; T4 = 2 years after childbirth

Among the total sample, participants were excluded if their questionnaires were not completed at all or not submitted in time. T1 questionnaires submitted before birth, T2 questionnaires handed in before 16 weeks postpartum, and T3 questionnaires handed in between the 13th and 14th months postpartum were considered timely. Additional exclusion criteria for this study included being a female partner, multiple births, and death of the child before reaching the age of 14 months. Follow-up rates and exclusion of participants from the total sample according to the exclusion criteria are detailed in Fig. 1. After these exclusions of participants, the final sample consisted of 2,529 participants, i.e., 1,544 mothers and 985 fathers (see Fig. 1).

### Measures

#### Main variables

Prenatal anxiety symptoms were assessed mainly during the last trimester of pregnancy in mothers and fathers via the German version of the anxiety subscale of the Symptom Checklist-90-Revised (SCL-90-R; [70, 71]). The SCL-90-R is a frequently used screening instrument for psychopathological symptoms. The participants rated 10 items, such as “trembling” or “feeling fearful,” on a 5-point Likert scale ranging from 0 (not at all) to 4 (very strongly), indicating the extent to which they experienced these symptoms in the past seven days. A high total score (range: 0–40) is an indicator of high levels of anxiety. The reliability of the scale was acceptable (mothers: Cronbach’s α =.75; fathers: Cronbach’s α =.78).

Child development was assessed 14 months after childbirth. The measurement tool was the Ages & Stages Questionnaire (ASQ-3) [72]. It is a recommended screening tool for developmental delays [73]. The questionnaire consists of 30 questions referring to the child’s milestone attainment. They are divided into five developmental domains: fine motor, gross motor, personal-social, communication, and problem solving. All questions were parent-completed on a 3-point-Likert-scale, ranging from 10 (yes), 5 (sometimes), to 0 (not yet). The total score, which was used for the analyses (range: 0–300), is calculated by summing up the cumulative scores of all five domains. A higher score represents more advanced development. The reliability of the scale was good (mothers: Cronbach’s α for =.83; fathers: Cronbach’s α =.85).

Parent-child bonding was assessed around eight weeks postpartum using the German version of the validated self-report Postpartum Bonding Questionnaire (PBQ) [74, 75]. The German version of the PBQ was provided by the Marcé society (https://marce-gesellschaft.de/materialien/). The PBQ was selected because it is a widely used tool for assessing parent-child bonding in research. Its validity was recently confirmed in a systematic review comparing all available parent-report measures of postpartum bonding, in which the PBQ received the highest psychometric ratings [76]. It includes 25 items, describing emotional reactions toward the baby, belonging to four subscales: “impaired bonding” (12 items), “rejection and anger” (7 items), “anxiety about care” (4 items), and “risk of abuse” (2 items). Mothers and fathers are asked to complete the questionnaire while they think of the most difficult time with their child. The items are rated on a 6-point-Likert scale, ranging from 0 (never) to 5 (always). All item ratings were summed up (range: 0–125), with a higher score indicating poorer parent-child bonding. The cutoff score for clinically relevant bonding impairment is at a score of more than 25 [74]. The tool was developed for assessing maternal bonding but can also be used for fathers. There is no validated paternal version of the PBQ, but it has been used in previous studies to measure the father-child bonding [52, 77, 78]. This is also widely accepted and supported by a recent concept paper on father-child bonding [51]. The reliability of the scale was good (mothers: Cronbach’s α =.89; fathers: Cronbach’s α =.85).

#### Confounders

The selection of potential confounders was based on the literature. The following variables have been shown to be associated with child development and/or prenatal anxiety symptoms: pregnancy complications [79, 80], breastfeeding [81, 82], child sex [27], preterm birth [60, 83], child-related birth complications [84], prenatal social support [19, 85], postnatal partnership satisfaction [86], parental education [87], COVID-19 pandemic exposure [88–90], and postnatal depression symptoms [44, 48].

Pregnancy complications, e.g., bleeding or hypertension, were assessed around eight weeks postpartum using maternal information, based on their maternity records [91]. An index was created indicating the number of complications that occurred (0,1,2, and ≥ 3). Information on preterm birth (i.e., childbirth before the 37th week of pregnancy) and child sex were retrieved from the T2 questionnaire. Child-related birth complications, e.g., pathological heart sounds or green amniotic fluid were also assessed as index variables (0,1,2, and ≥ 3) around eight weeks after childbirth, on the basis of maternity records. Current breastfeeding status was measured as a dichotomized variable around eight weeks after childbirth. For the variables pregnancy complications, breastfeeding, child sex, preterm birth, and child-related birth complications, only mothers’ data were used for all analyses. Fathers’ answers were used when their female partners’ information was missing.

Perceived social support in mothers and fathers was measured mainly during the last trimester of pregnancy with the short version of the German Social Support Questionnaire (“Fragebogen zur Sozialen Unterstützung”; F-SozU-14) [92]. It consists of 14 items, on a scale ranging from 0–5, resulting in a maximum score of 70. The mean value of all item scores was used for the analyses, which can be between 0 and 5. The scores were included as a metric variable. No official cut-off score was defined in the validation paper. The reliability of the scale was excellent (mothers: Cronbach’s α =.92; fathers: Cronbach’s α =.92).

Partnership satisfaction of mothers and fathers was assessed around eight weeks postpartum, using the short version of the Partnership Questionnaire (“Kurzform des Partnerschaftsfragebogens”; PFB-K) [93]. The total score (range: 0–27) consists of the sum of nine, four-point-Likert scale items. The scores were included as a metric variable. A cut-off score of approximately 12 is being discussed. The reliability of the scale was acceptable (mothers: Cronbach’s α =.80; fathers: Cronbach’s α =.78). The dichotomous variable education, defined as a minimum of ten years of school education or more, was assessed at T1. Given that the study was conducted partly during the COVID-19 pandemic, COVID-19 pandemic exposure was also controlled for. These variables were accounted for on the basis of the date of completion of T3, i.e., when participants reported the outcome variable of general child development. The participants were grouped into 12 different phases of the pandemic and relating establishment and easement of restrictions in Germany [94–96]. Postnatal depression symptoms in mothers and fathers were assessed around eight weeks postpartum using the German version of the Edinburgh Postnatal Depression Scale (EPDS) [97, 98]. It consists of 10 items capturing symptoms of depression during the previous week, each rated on a four-point Likert scale. The total score, ranging from 0–30, was used for analyses. The scores were included as a metric variable. The cut-off scores are < 10 for no depression, 10–13 for probable minor depression, and > 13 for probable major depression*.* The reliability of the scale was acceptable (mothers: Cronbach’s α =.80; fathers: Cronbach’s α =.80).

### Data analysis

**Fig. 2:**
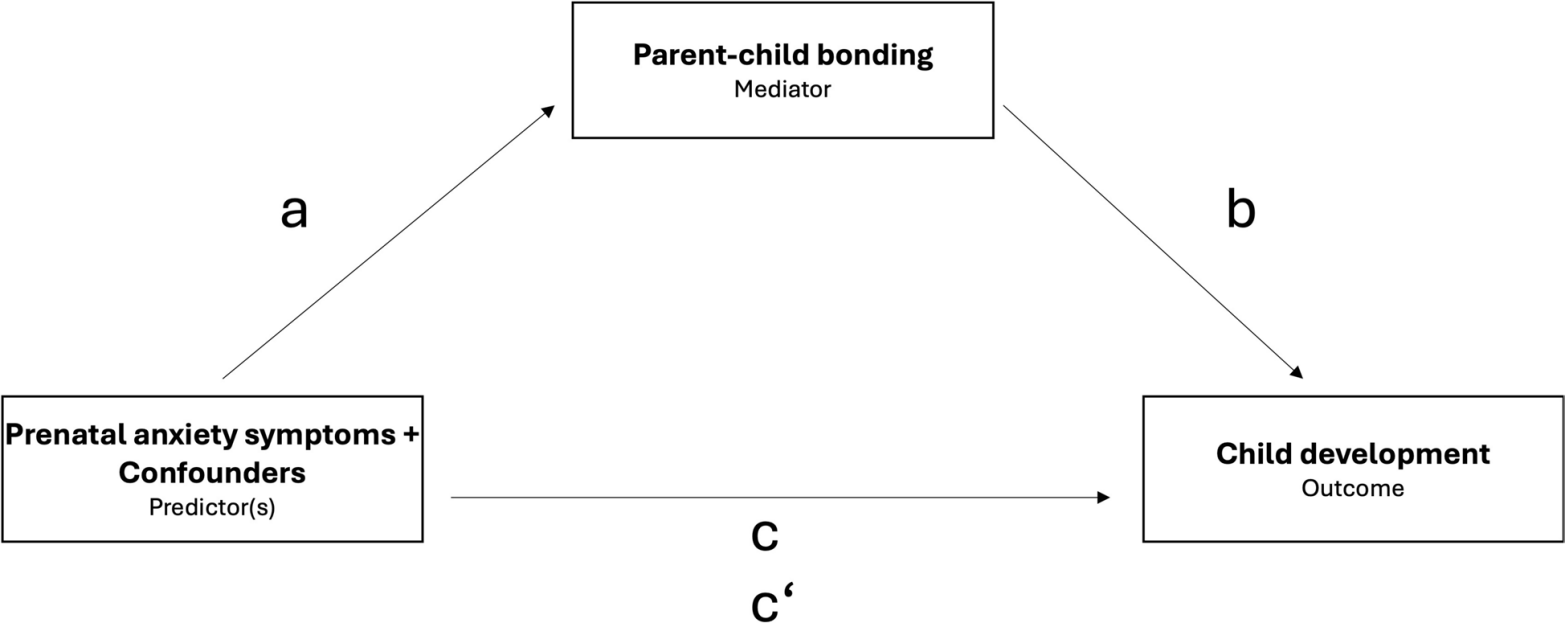
Mediation analysis a = pathway between predictor and mediator; b = pathway between mediator and outcome; c = total effect (pathway between predictor and outcome, without the mediator considered in the model; c’ = direct effect (pathway between predictor and outcome, with the mediator considered in the model)

IBM SPSS statistics 28 [99] was used for all the statistical analyses, which were conducted separately for mothers and fathers. If less than 20% of the metric variables were missing, the data were supplemented by mean imputation. This approach was chosen to minimize the number of excluded participants and thereby maximize the statistical power. Low rates of missing values for metric variables, ranging from 0.8% (child development—gross motor skills) to 2.7% (parent-child bonding), justified the use of this simple imputation method. Participants were excluded listwise per analysis if more than 80% of a psychometric variable’s items or a dichotomous variable’s data were missing. Consequently, sample size varied across analyses based on included variables. Descriptive analyses were conducted for the main variables, confounders, and additional socioeconomic factors. T-tests were used to investigate differences between mothers and fathers. Since the absence of outliers is a prerequisite for nearly all calculations, every variable was examined for univariate extreme values, using boxplots, and for multivariate outliers, using the Mahalanobis distance. Sensitivity analyses were carried out, which did not change the results; therefore, all participants were included in the analyses. The specific prerequisites of mediation analyses which are linearity, standard deviation of the residuals, homoscedasticity, no autocorrelation, and chronological succession of predictor, mediator, and outcome were tested. All were met, except for homoscedasticity. Therefore bootstrapping, with 5,000 iterations was used to compensate for this. Multicollinearity is not a prerequisite for mediation analysis [100], but when present between predictors and confounders, it can hinder interpretability. However, all VIF values in Model 3 (which includes all confounders) were below 2, indicating no multicollinearity concerns. Attrition analyses and correlation analyses were performed, also using bias-corrected accelerated (BCa) bootstrapping with 5,000 iterations. Mediation analysis (see Fig. 2) was conducted with the SPSS modeling tool PROCESS, Model 4, created by Hayes [101]. Model 1 included only the main variables. In Model 2, additional predictors were included, namely confounders that were significantly correlated with general child development. Postnatal depression symptoms however, were included separately in Model 3. This approach was chosen because of their high rate of comorbidity with anxiety symptoms [102] and their strong association with poor parent-child bonding [44, 48] and child development [103]. This allowed an investigation of the distinct influence of prenatal anxiety symptoms. Bootstrapping with 5,000 iterations was used to compensate for violations of the prerequisites, and the results were considered statistically significant when *p* <.05, or when the confidence interval did not include 0. To evaluate whether the associations differ between mothers and fathers the calculated standardized beta coefficients of the mediation analysis were compared descriptively. The manuscript was partially translated and stylistically improved using ChatGPT, DeepL, and Curie.

## Results

### Descriptive analyses

The final sample consisted of 2,529 participants, i.e., 1,544 mothers and 985 fathers. The characteristics of the sample are displayed in Table 1. The mothers’ mean age was 30.2 years (SD = 3.9) and the fathers’ mean age was 32.6 years (*SD* = 4,9). Most parents were expecting their first child (mothers: 81.1%; fathers: 81.4%), almost all were in a committed relationship (mothers: 98.9%; fathers: 99.9%), and German citizenship was held by the vast majority (mothers: 96.9%; fathers: 98.7%). Ten or more years of school education were completed by 79.6% of the mothers and 75.3% of the fathers. Nearly all mothers were breastfeeding their children eight weeks postpartum, which is a comparatively high number [104]. The rate of preterm birth (4.8%) was rather low compared to the German average (8.6%) [105]. The level of education in the final sample was high compared to that in the general population in Dresden [106]. In addition, levels of postnatal depression symptoms were lower than average [107, 108]. Mothers’ average SCL-90-R score (*M* = 2.52; *SD* = 3.01) was significantly higher than that of fathers (*M* = 1.73; *SD* = 2.65; *t* = 6.93; *p* = <.001; *d* =.28). The mean scores of the ASQ-3 varied only slightly between mothers and fathers (mothers: *M* = 226.90; *SD* = 42.38; fathers: *M* = 222.13; *SD* = 44.38). Nevertheless, the difference was statistically significant (*t* = 2.65; *p* =.008 *d* =.11), which means that mothers’ ratings of their child’s development were slightly better than those of fathers. The average PBQ score was nearly equal for mothers (*M* = 12.96; *SD* = 9.91) and fathers (*M* = 12.60; *SD* = 8.2), and they did not differ significantly (*t* = 0.94; *p* =.313, *d* =.04). The average levels of postnatal depression symptoms of mothers (*M* = 5.68; *SD* = 3.8) were significantly higher than those of fathers (*M* = 3.57; *SD* = 3.33; *t* = 14.61; *p* = <.001, *d* =.58). T1 questionnaires were returned from the 06/27/2017 to the 10/31/2020. T2 questionnaires were returned from the 29/09/2017 to the 22/01/2021. T3 questionnaires were returned from the 20/09/2018 to the 17/01/2022. Nearly half of the participants reported child development (T3) before the beginning of the COVID-19 pandemic, at the location of the study in Dresden, Germany, i.e., before 09.03.2020 (mothers: 41.1%; fathers: 42.9%). Detailed descriptions regarding the participant completion rates for T3 during different phases of the COVID-19 pandemic can be provided upon request.

**Table 1:** Sample characteristics

		Mothers (*n* = 1,544)	Fathers (*n* = 985)
		*n* (%) or *M* ± *SD*; Range	
Age^a^		30.2 ± 3.9; 15–43	32.6 ± 4.9; 20–56
Expectant first time parent	Expecting first child	1241 (81.1%)	778 (81.4%)
	Already having children	290 (18.9%)	178 (18.6%)
Partnership status^a^	Yes	1518 (98.9%)	974 (99.9%)
	No	17 (1.1%)	1 (0.1%)
German citizenship^a^	Yes	1496 (96.9%)	972 (98.7%)
	No	48 (3.1%)	13 (1.3%)
Education^a^	≤ 10 years	315 (20.4%)	242 (24.7%)
	> 10 years	1228 (79.6%)	736 (75.3%)
Academic degree	Academic degree	892 (58.0%)	564 (58.1%)
	No academic degree	642 (42.0%)	407 (41.9%)
Employment status during pregnancy^a,d^	Working full time (n, %)	724 (47.1%)	824 (83.9%)
	Working part time (*n*, %)	250 (16.3%)	79 (8.0%)
Mean week of pregnancy^a^		29.5 ± 6.5; 8–41	29.6 ± 6.5; 8–41
Child’s mean age in weeks at T2^b^		8.6 ± 2.2; 1–24	8.9 ± 2.2; 3–21
Child’s mean age in months at T3^c^		13.7 ± 0.5; 13–14	13.8 ± 0.4; 13–14
Prenatal anxiety symptoms (SCL-90-R)^a^		2.52 ± 3.01	1.73 ± 2.65
General child development (ASQ-3)^c^		226.90 ± 42,38	222.13 ± 44,38
Parent-child bonding (PBQ)^b^		12.96 ± 9.91	12.60 ± 8.2
Pregnancy complications^b^	0	875 (56.7%)	-^e^
	1	459 (29.7%)	
	2	159 (10.3%)	
	≥ 3	51 (3.3%)	
Breastfeeding^b^	No	110 (7.2%)	-^e^
	Yes	1410 (92.8%)	
Child sex^b^	Female	788 (51.6%)	-^e^
	Male	740 (48.4%)	
Preterm birth^b^	No	1470 (95.2%)	-^e^
	Yes	74 (4.8%)	
Child-related birth complications^b^	0	1013 (65.7%)	-^e^
	1	430 (27.8%)	
	2	84 (5.4%)	
	≥ 3	17 (1.1%)	
Social support (F-SozU-14)^a^		4.34 ± 0.56	4.22 ± 0.62
Partnership satisfaction (PFB-K)^b^		20.43 ± 4.32	19.64 ± 4.09
Postnatal depression symptoms (EPDS)^b^		5.68 ± 3.8	3.57 ± 3.33

Total sample number varies due to missing data; SCL-90-R, Symptom Check List 90 Revised; PBQ, Postpartum Bonding Questionnaire; ASQ-3, Ages and Stage Questionnaire-3; F-SozU-14, Short version of the Social Support questionnaire (German: Fragebogen zur Sozialen Unterstützung); PFB-K, Short Version of the Partnership questionnaire (German: Kurzform des Partnerschaftsfragebogens); EPDS, Edinburgh Postnatal Depression Scale; ^a^T1 during late pregnancy, ^b^T2, eight weeks postpartum, ^c^*T3* 14 months postpartum, ^d^Several other options regarding current employment status were given in the questionnaire, e.g., studying, unemployed, employment prohibition, etc., therefore percentages do not add up to 100%, ^e^The calculations were based only on information from mothers

### Correlation analyses

To have a homogeneous, comparable set of variables, every confounder that had a significant association with general child development in either mothers or fathers, was included in the mediation analyses. This was the case for pregnancy complications (mothers: *r* =-0.05; *p* =.040), breastfeeding (mothers: *r* = 0.10; *p* = < 0.001; fathers: *r* = 0.08; *p* = 0.027), child sex (mothers: *r* =-0.08; *p* = < 0.002 fathers: *r* = −0.15; *p* = < 0.001), preterm birth (mothers: *r* = -0.19; *p* = < .001; fathers: *r* = −0.17; *p* < 0.001), social support (mothers: *r* =0.07; *p* = .012); fathers: *r* = −0.14; *p* = .007), partnership satisfaction (mothers: *r* = 0,72; *p* <.001), education (mothers: *r* = 0.05; *p* =.043), postnatal depression symptoms (mothers: *r* =-0.06; *p* =.036; fathers: *r* = −0.14; *p* = < 0.001), and COVID-19 phases 5 (fathers: *r* =0.07, *p* =.045) and 6 (fathers: *r* = −0.08, *p* =.020), which are equivalent to the second wave. All correlation effects were small. The intercorrelations between all the study variables are displayed in Table 2. Detailed information regarding the correlations of the different phases of COVID-19 pandemic exposure with general child development can be found in the supplementary material.

**Table 2:** Intercorrelations between study variables for mothers (*n* = 1,513) and fathers (*n* = 809)

Study variables	1	2	3	4	5	6	7	8	9	10	11	12
1 Prenatal anxiety symptoms^a^ (T1)	–	.13^***^	-.03	.03	-.02	.01	.01	.02	-.22^***^	-.07	-.03	.38^***^
2 Parent-child bonding^b^ (T2)	.16^***^	–	-.13^***^	-.01	.00	-.02	-.04	-.01	-.19^***^	-.20^***^	.13^***^	.40^***^
3 General child development^c^ (T3)	-.03	-.08^**^	–	-.04	.08^*^	-.15^***^	-.17^***^	-.02	.14^***^	.07	.01	-.14^***^
4 Pregnancy complications (T2)	.07^**^	-.01	-.05^*^	–	-.03	.04	.26^***^	.01	.01	-.00	-.02	.06
5 Breastfeeding (T2)	-.02	-.01	.10^**^	-.03	–	.02	-.14^***^	.08^*^	-.02	-.01	.14^***^	-.09^*^
6 Child sex (T2)	-.03	-.04	-.08^**^	-.01	.02	–	-.00	.04	.05	.03	-.07	.01
7 Preterm birth (T2)	.02	.03	-.19^**^	.23^**^	-.10^**^	.01	–	-.06	.03	-.05	-.01	.02
8 Child-related birth complications (T2)	.01	.03	-.02	.07^**^	.02	.03	-.02	–	.01	.03	.02	-.03
9 Social support (T1)	-.20^***^	-.17^***^	.07^*^	-.04	.00	.04	-.02	-.01	–	.36^***^	.03	-.22^***^
10 Partnership satisfaction^e^ (T2)	-.09^**^	-.11^**^	.07^**^	.02	-.04	.04	-.06^*^	-.01	.30^***^	–	.01	-.23^***^
11 Education (T1)	-.05^*^	.14^***^	.05^*^	-.02	.19^***^	-.02	-.02	.04	.07^*^	-.01	–	-.01
12 Postnatal depression symptoms^f^ (T2)	.34^***^	.43^**^	-.06^*^	.11^***^	-.06^*^	-.02	.06^*^	.04	-.24^***^	-.15^***^	-.04	–

The table is mirrored along the diagonal centerline, with the maternal values positioned below and the paternal values above. Pearson’s correlation coefficient *r;* two-tailed test; T1, during late pregnancy; T2, eight weeks postpartum; T3, 14 months postpartum

^*^p <.05; ^**^p <.01; ^***^p <.001

^a^*SCL-90-R* Symptom Check List 90 Revised

^b^*PBQ* Postpartum Bonding Questionnaire

^c^*ASQ-3* Ages and Stage Questionnaire-3

^d^*F-SozU-14* Short version of the Social Support questionnaire (German: Fragebogen zur Sozialen Unterstützung)

^e^*PFB-K* Short Version of the Partnership questionnaire (German: Kurzform des Partnerschaftsfragebogens)

^f^*EPDS* Edinburgh Postnatal Depression Scale

### Attrition analysis

Attrition analyses were conducted to determine whether completers (final sample) and noncompleters (participants who completed only T1 and met one of the exclusion criteria) differed significantly in terms of socioeconomic factors and prenatal anxiety symptoms. Maternal completers had significantly more often more than 10 years of schooling (78.2% vs. 67.0%; χ^2^ = 16.19; *p* = <.001). The same was observed among fathers, with 75.3% of completers having more than 10 years of school education, compared to 56.7% of the noncompleters (χ^2^ = 24.29; *p* = <.001). Additionally, male completers held German citizenship significantly more often than non-completers (98.7% vs. 96.4%; χ^2^ = 4.61; *p* =.032). Results can be found in Tables 3 and 4.

**Table 3:** Attrition analyses for dichotomous variables by chi-square-test

**Variable**	**Mothers** (C: *n* = 1544; NC: *n* = 200; Missing = 483)					**Fathers** (C: *n* = 985; NC: *n* = 166; Missing = 467)				
	***n***^***a***^		**χ**^**2**^	***df***	***p***	***n***^***a***^		**χ**^**2**^	***df***	***p***
Parity	C: NC:	1531 200	3.47	1	.062	C: NC:	956 162	2.08	1	.149
Partnership status	C: NC:	1535 200	0.24	1	.625	C: NC:	975 166	0.17	1	.680
German citizenship	C NC:	1544 200	1.97	1	.160	C: NC:	985 166	4.61	1	.032
School Education	C: NC:	1543 197	16.19	1	<.001	C: NC:	978 164	24.29	1	<.001
Academic degree	C: NC:	1537 196	5.18	1	.023	C: NC:	971 165	22.59	1	<.001
Fulltime employment	C: NC:	1538 199	2.44	1	.118	C: NC:	982 166	1.77	1	.183

C = Completers, i.e., participants which completed questionnaires T1, T2, and T3 in time; NC = Noncompleters, i.e., participants who only completed T1 in time; two-sided significance testing; T1 = during late pregnancy; T2 = eight weeks postpartum; T3 = 14 months postpartum

^a^*n* slightly varies due to missing data for some variables

**Table 4:** Attrition analyses for metric variables by t-test

Variable	*n*^*a*^		*t*	*df*	*p*	Mean difference	*BCa 95% CI*
Mothers (C: *n* = 1535; NC: *n* = 200)							
Age	C: NC:	1531 199	−1.02	1728	.307	−0.304	[−0.886; 0.279]
Prenatal anxiety symptoms	C: NC:	1535 200	1.78	234	.077	0.487	[−0.054; 1.029]
Fathers (C: *n* = 976; NC: *n* = 165)							
Age	C: NC:	976 165	−0.70	248	.429	−0.285	[−0.993; 0.423]
Prenatal anxiety symptoms	C: NC:	976 165	−0.06	1138	.953	−0.013	[−0.449; 0.423]

C = Completers, i.e., participants which completed questionnaires T1, T2, and T3 in time; NC = Noncompleters, i.e., participants who only completed T1 in time; T1 = during late pregnancy; T2 = eight weeks postpartum; T3 = 14 months postpartum; prenatal anxiety symptoms was measured by SCL-90-R = Symptom Check List 90 Revised; two-sided significance testing

^a^*n* slightly varies due to missing data for some variables

### Mediation analysis

The direct effect between prenatal anxiety symptoms and child development was not significant. But higher levels of prenatal anxiety symptoms predicted poorer parent-child bonding, which in turn predicted impaired child development. However, this mediation effect disappeared when postnatal depression symptoms were included.

In Model 1 (see Fig. 2), which included only the main variables, no significant direct prospective association between prenatal anxiety symptoms and general child development was found (c’; mothers: β = −.025; *p* =.363; fathers: β = −.002;* p* =.956). However, higher levels of prenatal anxiety symptoms significantly predicted poorer parent-child bonding eight weeks postpartum (a; mothers: β =.188; *p* <.001; fathers: β =.172; *p* <.001), which in turn significantly predicted poorer general child development 14 months postpartum (b; mothers: β = −.075; *p* =.009; fathers: β = −.146; *p* <.001). Accordingly, the indirect effect was also significant (ab; mothers: β = −.014; *BCa 95% CI* = [−0.417; −0.049]; fathers: β = −.025; *BCa 95% CI* = [−0.951; −0.236]), which means that poorer parent-child bonding was a significant mediator for the association between higher levels of prenatal anxiety symptoms and impaired general child development in Model 1.

In Model 2 (see Fig. 2), all confounders (except for postnatal depression symptoms) which were significantly related to general child development in the preceding correlation analyses were added as predictors. We found results similar to those of Model 1. Again, the direct association was not significant (c’; mothers: β = −.011; *p* =.686; fathers: β =.043; *p* =.264). Pathway a remained statistically significant (mothers: β =.154; *p* <.001; fathers: β =.152; *p* = <.001) as did pathway b (mothers: β = −.083; *p* =.007; fathers: β = −.117; *p* =.002) and the indirect path (ab; mothers: β = −.013; *BCa 95% CI* = [−0.376; −0.049]; fathers: β = −.019; *BCa 95% CI* = [−0.789; −0.104]). This means, that also when controlling for several confounders, parent–child-bonding significantly mediated the association between prenatal anxiety symptoms and general child development 14 months postpartum.

**Fig. 3:**
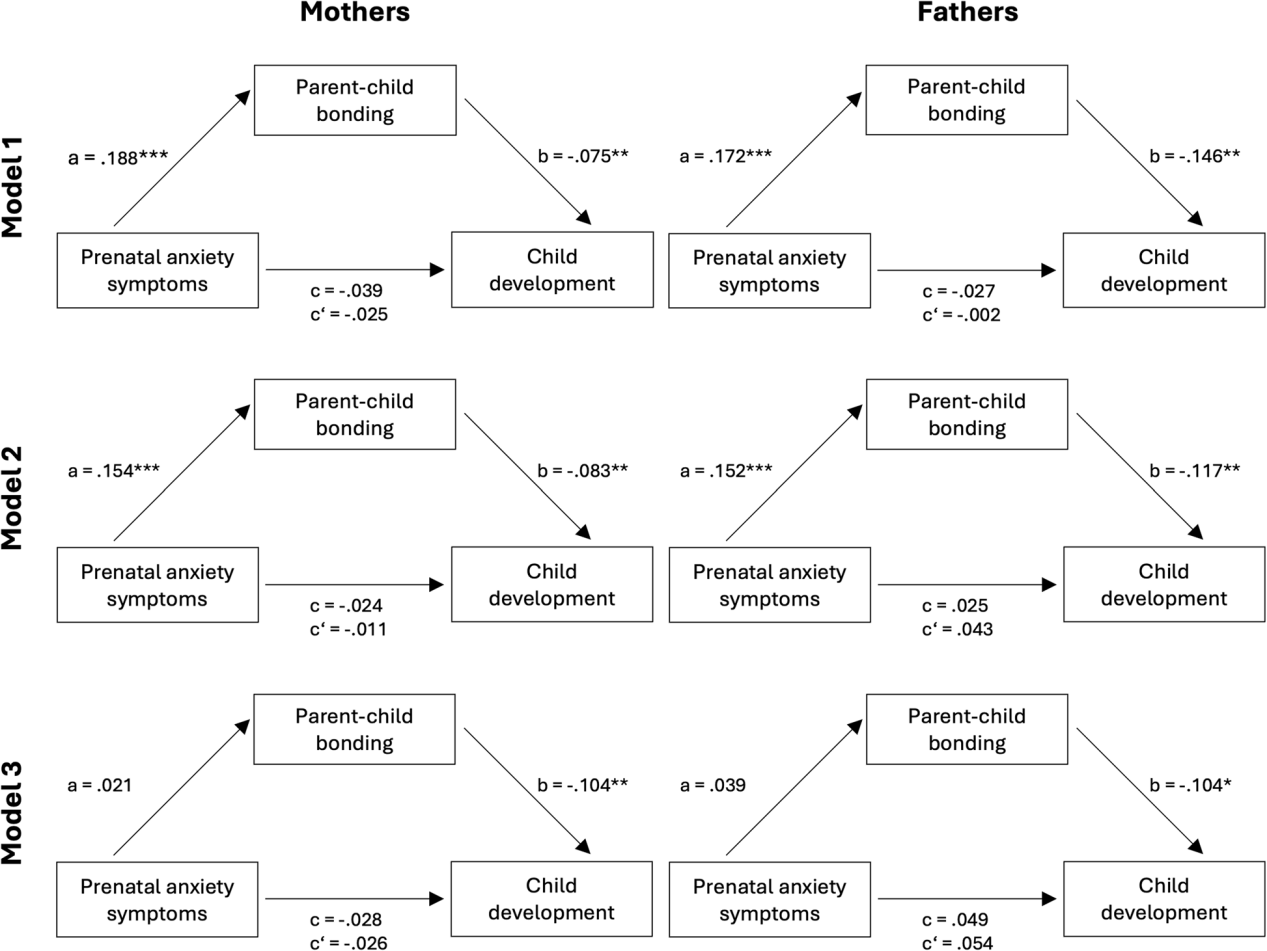
Standardized regression coefficients of the mediation a x b = indirect effect; c = total effect; c’ = direct effect; Model 1 included the main variables: Prenatal anxiety symptoms were assessed by the SCL-90-R = Symptom Check List 90 Revised; Parent-child bonding was assessed by the PBQ = Postpartum Bonding Questionnaire; Child development was assessed by the ASQ-3 = Ages and Stage Questionnaire-3; Model 2 included several confounders: pregnancy complications, child sex, preterm birth, birth complications, education, Social support, which was assessed by the F-SozU-14 = Short version of the Social Support questionnaire (German: Fragebogen zur Sozialen Unterstützung), and partnership satisfaction which was assessed by the PFB-K = Short Version of the Partnership questionnaire (German: Kurzform des Partnerschaftsfragebogens); Model 3 additionally included postnatal depression symptoms, which were assessed by the EPDS = Edinburgh Postnatal Depression Scale. *p <.05; **p <.01; ***p <.001

In Model 3 (see Fig. 3), postnatal depression symptoms eight weeks postpartum were added as an additional confounder. Again, the direct pathway (c’; mothers: β = −.026; *p* =.374; fathers: β =.054; *p* =.167) was not significant. However, prenatal anxiety symptoms did no longer significantly predict parent-child bonding (a; mothers: β =.021; *p* =.462; fathers: β =.039; *p* =.367). Instead, postnatal depression symptoms highly significantly predicted impaired child development (mothers: β =.421; *p* = <.001; fathers: β =.360; *p* = <.001). However, parent-child bonding remained a significant predictor for general child development (b; mothers: β = −.104; *p* =.002; fathers: β = −.104; *p* =.012). Nevertheless, the indirect pathway (ab; mothers: β = −.002; *BCa 95% CI* = [−0.137; 0.053]; fathers: β = −.004; *BCa 95% CI* = [−0.354; 0.098]), was no longer significant, meaning that parent-child bonding was no significant mediator when postnatal depression symptoms were controlled for. Tables with the exact results of all the mediation analyses and a detailed table of pathway a with all confounder values can be found in the supplementary material. Overall, the standardized regression coefficients were small, indicating small effect sizes [109].

The goodness of fit of the models was assessed by the coefficient of determination (R2). According to Cohen’s rule of thumb (1988), the goodness of fit for Model 1, which included only the main variables, was weak (mothers: R2 =.006; fathers: R2 =.021). Model 2, which included all other confounders except postnatal depression symptoms, had an improved but still weak goodness of fit (mothers: R2 =.062; fathers: R2 =.095). The inclusion of postnatal depression symptoms in Model 3 did not significantly affect the goodness of fit (mothers: R2 =.062; fathers: R2 =.099). In general, the variance in outcomes is better explained by the models for fathers.

## Discussion

This study investigated the mediation effect of postnatal parent-child bonding at eight weeks postpartum on the association between maternal and paternal prenatal anxiety symptoms with general child development at 14 months postpartum. The sample on which the analyses were conducted is a population-based sample from Dresden, Germany and the surrounding area. Most of the participating parents were expecting their first child, were mostly well-educated, healthy, and in stable employment and in long-term relationships. In this study no direct effect between prenatal anxiety symptoms in mothers and fathers and general child development was found. However, higher prenatal anxiety symptoms significantly predicted poorer parent-child bonding eight weeks postpartum in mothers and fathers, also when several perinatal confounders, i.e. pregnancy complications, breastfeeding, child sex, preterm birth, child-related birth complications, education, social support, partnership satisfaction, and COVID-19 pandemic exposure were controlled for. However, the association did not remain significant when postnatal depression symptoms were additionally controlled for. In turn, poor parent-child bonding in mothers and fathers predicted impaired general child development at the age of 14 months, also when controlling for all confounders, including postnatal depression symptoms. Hence, parent-child bonding significantly mediates the association between prenatal anxiety symptoms and general child development, but only when postnatal depression symptoms are not controlled for. The standardized regression coefficients did not differ greatly between mothers and fathers, and the significance was the same for all associations.

### The prospective association of prenatal anxiety symptoms with general child development

The direct associations between prenatal anxiety symptoms and general child development were not statistically significant, in all three models, which contradicts a considerable body of research. Review papers by Delagneau et al. [24], Quagliato et al. [25], and Rees et al. [26], reported an association between prenatal anxiety symptoms in mothers and the socioemotional and cognitive child development domains. However, the overall effect sizes in these reviews were small, indicating a weak association between the two variables. With respect to fathers, the body of research on this specific association is considerably smaller, and the results are contradictory. Two studys, by Bekkhus et al. [31] and Kvalevaag et al. [32] suggest a significant association whereas another study by Rogers et al. [33] could not find this effect.

Several factors may account for the lack of a significant association between prenatal anxiety symptoms and general child development in this study. First, this study assessed general child development, including fine motor and gross motor development, next to the domains of communication, personal-social, and problem solving (representing socioemotional and cognitive development domains). The studies quoted above, which have reported significant associations, have investigated only socioemotional and cognitive child development outcomes [24–26, 31, 32]. The results of this study could indicate that the negative influence of prenatal anxiety symptoms does not extend to the motor development domain. This conclusion is supported by other studies that have examined the association between prenatal anxiety and various domains of child development, finding a significant association between prenatal anxiety symptoms and socioemotional domains of child development, but not motor development [33], or an association between prenatal depression symptoms and motor development, but no association with prenatal anxiety symptoms [110]. These findings are further supported by the theory of fetal programming, which suggests a potential mechanism underlying this association. The literature on fetal programming often refers to the maternal stress response resulting in dysregulation of the maternal HPA axis, which in turn may influence the calibration of the embryo’s HPA axis toward hyperreactivity [37, 111]. A hyperreactive HPA axis can lead to hypervigilance and easily distracted attention or anxiety [112]. This will affect socio-emotional developmental domains rather than motor developmental domains.

Second, levels of experienced prenatal anxiety were overall low in this community sample, lower than in another population-based study that used the same anxiety scale for assessment [113]. It is possible that only a greater severity of symptoms leads to measurable negative effects on child development. This phenomenon has been described previously by Parfitt et al. [114] regarding the negative effects of perinatal depression symptoms. Non pathological levels of anxiety have even been found to be associated with better child development in a sample of exclusively healthy mothers in stable socioeconomic situations [28]. The authors suggested that moderate levels of maternal prenatal anxiety or stress and the corresponding intrauterine conditions (more frequent changes in maternal visceral sounds or temperature, moderate hypoxia, and higher cortisol levels) may lead to better stimulation of fetal development in healthy populations. Keim et al. [29] also reported nonlinear associations between motor development and receptive language ability with postnatal depression symptoms, trait anxiety, and perceived stress, which indicates as well that mild levels of anxiety symptoms do not necessarily entail impaired child development. A recent neurobiological review by Engel & Gunnar et al. [37] further supports this notion by arguing that the theory that a moderate amount of stress may be beneficial for children’s development and necessary for an optimal calibration of the HPA axis reactivity.

Third, a multitude of perinatal factors influence child development but are also related to prenatal anxiety symptoms [115]. Susceptibility to the negative influence of prenatal anxiety on child development might be increased by the prevalence of other risk factors and the presence of general difficulties [114, 116]. The sample of the current study however is generally rather privileged, socioeconomically stable, and mostly healthy [69]. For instance, rates of breastfeeding, preterm birth, postnatal depression symptoms, and education were more favorable than in the general population [104–108]. Also, completers had significantly better education than noncompleters in the final sample of this study. Many of these factors have been related to child development in previous research [48, 82, 83, 85–87]. These favorable circumstances may have buffered the negative effects of prenatal anxiety symptoms and potentially even supported child development [37, 38, 115].

Moreover, studies that also found no significant effect for this association may not have been published because it is more difficult to publish non-significant results. This is known as publication bias and may have led to an overestimation of the association between prenatal anxiety symptoms and child development in the existing reviews [117]. This notion is supported by the overall small effect sizes found in the systematic reviews and meta-analyses.

Finally, this study is not the first population-based study which could not replicate the commonly reported significant association between prenatal anxiety symptoms and child development [68, 110, 118]. These studies were similar to the present study in several ways. The samples in Sommer et al. [110] and Polte et al. [118] were similarly socioeconomically advantaged, and Sommer et al. [110] also assessed general child development. Another large population based study by Le Bas et al. [68] which conducted a similar mediation analysis, did not find a direct significant association between affective symptomatology and socioemotional child development. However, they found postnatal parent-child bonding to be a significant mediator for this association in mothers.

### The prospective association of prenatal anxiety symptoms with parent-child bonding

When only the main variables were included in the analyses and when controlling for confounders, higher maternal and paternal prenatal anxiety symptoms significantly predicted worse parent-child bonding. However, when postnatal depression symptoms were added to the model, the effect was no longer significant, instead now postnatal depression symptoms significantly predicted poorer parent-child bonding.

The results of this study regarding mothers correspond to the results of previous studies. Nath et al. [119] also reported that prenatal anxiety symptoms in mothers predict poorer postnatal mother-child bonding, but only when prenatal depression symptoms were not controlled for. These findings are further supported by two review papers by O’Dea et al. [48] and Tichelmann et al. [44] that reported that prenatal anxiety symptoms are generally associated with mother-child bonding, but perinatal depression is a stronger predictor.

Regarding the paternal association, existing research is sparse, and the findings of this study correspond only partially. De Cock et al. [56] also reported an association between higher levels of anxiety symptoms and worse father-child bonding. Other studies by Kerstis et al. [52] and Wells & Jeon [120] also reported evidence for an association between paternal postnatal depression symptoms and father-child bonding difficulties. In contrast, Nasreen et al. [121] did not find a significant association between prenatal anxiety symptoms and father-child bonding. Instead, they reported that depressed fathers showed poorer father-child bonding than anxious fathers.

The association of poorer parent-child bonding with higher levels of prenatal anxiety, and even more so with postnatal depression symptoms, may be explained in several ways. For example, repetitive negative thinking during pregnancy, which is a common symptom of affective mental health disorders, was found to be associated with poorer postnatal parent-child bonding [122]. This might be due to negative repetitive thoughts involving the child, or the parent being distracted by these thoughts from engagement with their child. More specifically, symptoms of anxiety during pregnancy, such as constant worrying and fearfulness [9] have been found to disturb the process of developing maternal emotional proximity towards the unborn child [40]. Poor prenatal parent-fetus bonding in turn is significantly associated with poor postnatal bonding in mothers and fathers [44, 123, 124].

Parent-child interactions, which are closely related to parent-child bonding [125] might also be relevant for the transmission of the effect. Interaction between caregivers and child already begins during pregnancy and increases in the postnatal period with touch, gestures, facial expressions, mutual gazes, and vocalization [60]. Maternal and paternal prenatal anxiety symptoms have been found to be associated with mother- and father-child interaction [40]. In a study by Parfitt et al. [114] even to a greater extent than postnatal mental health difficulties. For example, general anxiety symptoms during the last trimester have been associated with greater maternal intrusiveness eight months after childbirth [126]. However, another study by Nath et al. [119] could not replicate a significant association between prenatal anxiety symptoms and mother–child interaction.

Prenatal anxiety symptoms might also influence the hormonal balance of oxytocin, vasopressin, prolactin, and the sex hormones estradiol, progesterone, and testosterone which have been associated with influencing the onset and maintenance of maternal and paternal behavior [59, 127]. Regarding the establishment of parent-child bonding, especially oxytocin appears to play an important role [128–130]. However, research on the association between prenatal psychopathology and oxytocin levels is contradictory, with some studies finding an association with elevated and others with lower oxytocin levels, as described by Saxbe et al. [131].

But why do postnatal depression symptoms predict poorer parent-child bonding more strongly? O’Dea et al. [48] suggested that the difference in the strength of the associations between anxiety and depression symptoms they found in their review analysis may be due to the greater number of studies on perinatal depression. However, the results of the present study rather support the notion that depression symptoms are more strongly associated with parent-child bonding than prenatal anxiety symptoms, as the regression coefficient for postnatal depression is much higher than for prenatal anxiety.

The nature of depression symptomatology, such as a lack of pleasure and emotional unavailability due to a limitation to a negative range of emotion seem to hinder the development of a strong positive emotional bond toward the child even more strongly than prenatal anxiety symptoms [121, 132, 133]. Moreover, depression symptoms in the postnatal period may affect parental sensitivity towards the child, parenting skills, engagement with the child, and responsiveness to child cues [60, 62, 115], which are crucial for parent-child interactions [119]. Nakić Radoš et al. [134] reported that parental non-responsiveness mediated the associations between maternal and paternal postnatal anxiety and depression symptoms and poorer parent-child bonding. Another explanation may be that parents with symptoms of depression display a cognitive bias driven by selective attention to negative information, the misinterpretation of ambiguous details in a negative way, and an increased recall of negative memories [133]. Consequently, they may rate their emotional bonding towards their child more negatively than healthy or anxious parents.

Additionally, the difference in the timing of predictor assessment may have contributed to differences in predictive strength. Postnatal affective symptoms generally appear to have a stronger influence on parent-child bonding than prenatal affective symptoms, as concluded in the review by O’Dea et al. [48]. However, because of the simultaneous measurement of postnatal depression symptoms and parent-child bonding it is not possible to say whether postnatal depression symptoms preceded and caused the associated bonding difficulties, or vice versa.

Finally, just because postnatal depression symptoms are the stronger predictor of poorer parent-child bonding, this does not mean that the presence of prenatal anxiety symptoms is not relevant to the development of parent-child bonding. This becomes clear when considering the significance of the association between prenatal anxiety symptoms and parent-child bonding in the presence of 8 other perinatal confounders.

### The prospective association of parent-child bonding with general child development

Poor parent-child bonding significantly predicted impaired general child development for mothers and fathers, independent of all confounding factors, including postnatal depression symptoms. These findings are in line with previous research. A review paper by Le Bas et al. [57], summarized the first indications that the concept of postnatal mother-child bonding is associated with child development. However, they mainly included studies on prenatal mother-child bonding and the outcome of child temperament, and not child development in terms of milestone attainment. The outcome of milestone attainment in different developmental domains was assessed by Alhusen et al. [135], who reported a significant relationship with prenatal mother-child bonding, but only as long as postnatal depression was not controlled for. Two recent studies by Le Bas et al. [54] and Rusanen et al. [55] also found significant associations between poor postnatal mother-child bonding and socioemotional child development. Le Bas et al. [54] additionally investigated the associations with cognitive, language, and motor development, but reported that the effect sizes were much smaller for these developmental domains compared to socioemotional development. Rusanen et al. [55] controlled for postnatal depression symptoms, which did not have an influence on the significance of the association.

Research on this association investigating fathers is extremely sparse. But considering that the concept of mother-child bonding is usually assumed to be very similar to the concept of father-child bonding, as stated by the most recent concept analysis by Nakić Radoš et al. [43] and that fathers have become more invested in caretaking, as described in a German fathers report [65], it is not surprising that there was no difference in the significance of the association between parents in the present sample. A study by de Cock et al. [56], also found an association between poor mother- and father-child bonding and impaired child executive functioning at the age of two.

This study contributes evidence indicating that postnatal parent-child bonding may be related to general child development and not only to socioemotional and cognitive development, independent of postnatal depression symptoms. Several factors that have been discussed in the literature might influence the transmission of this effect and are briefly introduced in the following.

First of all, the child is fully dependent on their caregivers during the first months and years of life, when development is fastest and most susceptible to influences [136]. Therefore, the importance of parent-child bonding seems obvious. Good mother- and father-child bonding promotes positive and stimulating parent-child interactions and parenting skills [41, 51]. Stimulation, responsiveness, and early learning are essential for children’s development [17, 39]. Accordingly, parent-child interaction and parenting have been associated with language, cognition, motor, and social development [39, 58–61]. Little parental engagement and warmth, a lack of sensitivity, poor responsiveness, and intrusiveness are linked with impaired child development [55, 115]. Neglect and inconsistent behavior are the most damaging parent behaviors which can permanently affect brain structure and impair development [136]. Likewise, cognitively stimulating activities, physical care, emotional warmth, and sensitivity are correlated with better outcomes [58, 59]. Thereby the quality of the time spent together seems more relevant than the quantity [59].

Apart from influencing the social environment of the child, strong parent-child bonding also impacts the child at a biological level. The experience of nurturing care and strong parent-child bonding influences the activation and calibration of the HPA axis [22, 37], also because the stress system of the child cannot regulate itself during the first months of life [37]. Adverse early care and poor parent-child bonding can be a powerful stressor, resulting in a prolonged and more pronounced activation of the HPA axis, which additionally may leave the organism even more vulnerable to further adverse experiences [112]. At the same time, secure relationship experiences can buffer adverse influences on the stress system, through a reduction in HPA axis activation [37].

### Mediation effect of parent-child bonding

When including only the main variables and controlling for confounding factors, the indirect effect was significant. However, this significance was no longer observed once postnatal depression symptoms were added to the model. Hence, in alignment with Rucker et al. [137] and Zhao et al. [138], who asserted that the statistical significance of the indirect effect is sufficient for showing that a mediation effect is present, parent-child bonding significantly mediates the association between maternal and paternal prenatal anxiety symptoms and general child development when controlling for several confounders. These results are limited by the inclusion of postnatal depression, which renders the mediation effect non-significant, because the association between prenatal anxiety symptoms and postnatal parent-child bonding is no longer significant. Hence, postnatal depression symptoms appear to be a stronger predictor of poor parent-child bonding than prenatal anxiety symptoms.

These findings complement those of a study of Le Bas et al. [68], where a significant mediation effect of mother-child bonding for the association between maternal psychological distress (pre- and postnatal anxiety, depression, and stress) and children’s socioemotional development was found.

In the present study, the standardized regression coefficients were quite small, which was also the case in the study of Le Bas et al. [68]. Small, standardized regression coefficients generally indicate a weak association of the variables. However, the small size of the effect may also be due to the inclusion of generally healthy participants in the population-based samples of both studies. The effects are likely more pronounced in high-risk samples than in low-risk samples [38]. Le Bas et al. [68] further reported that these small effect sizes are indicators of the complex interplay of genetic, biological, and environmental pathways influencing child development. Given the healthy sample and the intricate interplay of various factors affecting child development during the perinatal period, the absence of large effects is not surprising.

### Comparison between mothers and fathers

Fathers showed lower levels of prenatal anxiety and postnatal depression symptoms than mothers, which is in line with previous findings by Leifermann et al. [8] and Kerstis et al. [52]. On average, they rated their children’s development worse than mothers. These findings also correspond to previous findings by Cepanec et al. [139] and might be further explained by fathers knowing their offspring less well, since they tend to spend less time with their children during the first year of life [140]. Furthermore, in this study father-child bonding did not differ significantly from mother-child bonding. This was surprising since several other studies by Edhborg et al. [141], Escribano et al. [142], and Kerstis et al. [52] reported that mother–child bonding was stronger than father-child bonding. The standardized regression coefficients of the mediation analyses were similar for mothers and fathers, and significance of the associations was the same. This is only partially in line with existing research as discussed above.

The results of this study contribute evidence that father-child bonding can be just as strong as mother-infant bonding and may be equally impaired by the prevalence of mental health issues during the perinatal period. Furthermore, the results show that father-infant bonding is as relevant for their child’s development as mother-infant bonding. These results contribute to filling the scientific knowledge gap regarding fathers in the perinatal period.

### The study in the context of the COVID-19 pandemic

Part of the data collection took place during the global COVID-19 pandemic. The pandemic affected social life and support, work situation, personal health, and health care, making it a strong stressor that increased prenatal anxiety symptoms in mothers [89, 90]. In turn, the societal restrictions implemented to mitigate the pandemic, such as the closure of social institutions and the restriction of social contact, may have affected children’s development. And the risk of developmental impairment increases with the quantity of adverse experiences due to the pandemic situation [88]. However, the focus of this work was not the source of anxiety, but rather the prevalence of anxiety symptoms and their predictive character on child development. The pandemic did not necessarily influence the predictive nature of anxiety but the negative impact on child development could have led to an overestimation of the associations. Therefore, it was controlled whether scores on the development scale were correlated with a particular phase of the COVID-19 pandemic. This was the case for two phases of the pandemic (time between the beginning of the second wave (10/09/2020) and the second lockdown (until 2/14/21) in fathers). Therefore, these two phases were included as confounders in the mediation analysis where they did not change the significance of the associations under investigation.

### Clinical implications

Addressing the emotional health of expectant parents has been neglected in obstetrics [143], and despite its prevalence and comorbidity with depression, perinatal anxiety disorders are often overlooked [144]. This study provides evidence for the importance of screening both parents for symptoms of anxiety and depression, both during pregnancy and postpartum. However, fathers may need to be approached more proactively, as cultural notions of masculinity and stigma may prevent them from expressing their emotional difficulties or seeking help themselves [145], and because they generally have less contact with the health care system during the perinatal period than mothers [51]. Screening is worthwhile because effective treatment options are available for mothers and fathers, with psychotherapy as first-line therapy [146]. However, there is a lack of prevention and treatment for fathers during the perinatal period [147], which needs to be changed in perinatal clinical care.

In addition, more attention should be paid to parent-child bonding, especially among parents with affective mental health problems. Given the serious consequences of poor bonding for child and family, early detection of poor bonding, for example with the PBQ [48], is important, also because good treatment options are available [47].

Parent-child bonding can be directly promoted by health care providers through adequate preparation for childbirth and the postpartum period, allowing physical proximity to the newborn, being aware of and addressing common life stressors, and promoting a positive emotional state at birth [41, 67, 148, 149]. Despite the many similarities between mother-infant bonding and father-infant bonding, fathers may need interventions tailored specifically for them. Additionally, it may be beneficial to more actively engage fathers, such as attending childbirth and parenting preparation classes, performing umbilical cord cutting, and increasing their involvement in childcare tasks [67].

Interventions for poor mother-infant bonding typically aim to increase mother-infant interaction and improve responsiveness to infant cues [48]. In addition to psychotherapy, interventions for poor bonding may include transcranial magnetic stimulation, hypnosis, and eye movement desensitization and reprocessing [41]. When parent-child bonding impairment occurs in the context of a mental health condition, it is advisable to address the mental health issue along with the bonding difficulties [44, 150]. This can take place in a variety of settings, such as the home environment or an outpatient clinic, and can be supported by video-based methods [151].

### Strengths and limitations

The primary strength of this study lies in its large sample size and prospective study design, allowing the investigation of longitudinal processes by evaluating the same cohort at multiple time points. To our knowledge this is the first longitudinal cohort study investigating the mediation role of postnatal parent-child bonding in the association between prenatal anxiety symptoms and general child development, considering the developmental domains of fine motor, gross motor, personal-social, communication, and problem solving, in both, mothers and fathers. For the assessment of the main variables only well validated and widely used measurement tools for anxiety symptoms, parent-child bonding, and general child development with good to excellent internal reliability were used [71, 76, 152]. Furthermore, this study took a wide variety of confounders into account, which is very important considering the complex interplay of relevant factors during the perinatal period.

However, some limitations should be noted. First, the sample of this study was rather privileged, socioeconomically well situated, healthy, and most participants were in stable long-term relationships [69]. These sample characteristics are common for epidemiological, longitudinal studies [153, 154] and for studies on perinatal mental health and child development [134]. However, this sample bias leads to limited generalizability to less privileged, less educated, or clinical samples, since effects in this healthy sample might underestimate the relevance for populations that are clinically more impaired and live in more difficult circumstances. Even though it is relevant for assessing perinatal interrelationships in low-risk samples, since this group makes up a large part of our population, future studies should try to recruit more diverse, averagely educated, and less privileged participants to improve generalizability. Due to the imputation strategy, there may be some bias in the values, and other imputation methods could emphasize robustness in future analyses. Moreover, all variables were assessed with self-rating tools, which are inherently subjective, unverifiable, and uncontrolled and might have led to bias, such as social desirability bias [155]. Mental health difficulties, parent-child bonding, and child development are subject to strong social norms, which might have led to socially more desirable responses. This effect may have led to an underestimation of the effect size if anxiety was reported less severe and parent-child bonding and child development were reported more favorably than reality. Since the investigated variables are influenced by other factors of the complex perinatal period, there are other variables that would have been interesting to include. This is also indicated by the small, standardized regression coefficients and the overall weak goodness of fit for all the models of the mediation analyses. In particular, the inclusion of prenatal parent-child bonding, prenatal depression symptoms, postnatal anxiety symptoms, and parent-child interaction would have enriched the understanding and helped to interpret the findings. Finally, it should be noted that due to the methodological limitations of quantitative research, establishing clear causal relationships for the studied associations remains challenging [143, 156].

## Conclusions

This large prospective cohort study revealed a significant mediation effect of parent-child bonding eight weeks after childbirth for the association between prenatal anxiety symptoms in mothers and fathers and general child development at the age of 14 months. This mediation effect remained significant also after the inclusion of diverse confounders. However, when postnatal depression symptoms were additionally controlled for, the effect was no longer significant. It can be concluded that mental health problems during pregnancy are relevant for the development of parent-child bonding, with prenatal anxiety symptoms having an effect independent of other perinatal factors but postnatal depression symptoms impacting bonding even more. Moreover, parent-child bonding seems to influence child development. Notably, there were no major differences between mothers and fathers. This underscores that fathers’ mental health and father-child bonding are equally important to mothers’ regarding child development. Consequently, it is important to screen for mental health and the quality of parent-child bonding in both parents [121]. Therapeutic measures should be initiated as quickly as possible when problems arise. A special unused potential, also for improving child development lies in the inclusion of fathers in clinical and scientific work.

## Supplementary Information

Supplementary Material 1.

## Data Availability

Data is provided within the manuscript or supplementary information files. Further information is available upon request.

## Abbreviations

T1: Measurement point during pregnancy
T2: Measurement point eight weeks after childbirth
T3: Measurement point 14 months after childbirth
ASQ-3: Ages and Stage Questionnaire-3
DREAM: DResdner Studie zu Elternschaft, Arbeit und Mentaler Gesundheit
EPDS: Edinburgh Postnatal Depression Scale
FSozU-14: Short version of the Social Support questionnaire
HPA axis: Hypothalamic-pituitary-adrenocortical axis
PBQ: Postpartum Bonding Questionnaire
PFB-K: Short version of the Partnership Questionnaire
SCL-90-R: Symptom Check List 90– Revised
