# Supplementary material for: The prospective association of prenatal anxiety symptoms in mothers and fathers with general child development 14 months postpartum and the mediating role of parent-child bonding: a mediation analysis within the longitudinal cohort study DREAM
Source: BMC Pregnancy Childbirth. 2025 Sep 9;25:931. doi: 10.1186/s12884-025-07846-z (PMC12421753; doi:10.1186/s12884-025-07846-z)
Supplement: Supplementary file 1 — Supplementary Material 1. [file 12884_2025_7846_MOESM1_ESM.docx]

Supplementary material

Supplementary material 1: Intercorrelations between general child development and COVID-19 pandemic-driven adversities for mothers

| **Study variables** | **1** | **2** | **3** | **4** | **5** | **6** | **7** | **8** | **9** | **10** | **11** | **12** | **13** | **14** |
| --- | --- | --- | --- | --- | --- | --- | --- | --- | --- | --- | --- | --- | --- | --- |
| 1 Child development | – |  |  |  |  |  |  |  |  |  |  |  |  |  |
| 2 Before COVID-19 | .007 | – |  |  |  |  |  |  |  |  |  |  |  |  |
| 3 Phase 1 | -.040 | -.186*** | – |  |  |  |  |  |  |  |  |  |  |  |
| 4 Phase 2 | -.012 | -.155*** | -.041 | – |  |  |  |  |  |  |  |  |  |  |
| 5 Phase 3 | .022 | -.136* | -.036 | -.030 | – |  |  |  |  |  |  |  |  |  |
| 6 Phase 4 | .006 | -.296*** | -.078** | -.065* | -.057* | – |  |  |  |  |  |  |  |  |
| 7 Phase 5 | .039 | -.231*** | -.061* | -.051 | -.045 | -.097*** | – |  |  |  |  |  |  |  |
| 8 Phase 6 | -.005 | -.228*** | -.060 | -.050 | -.044 | -.096*** | -.075** | – |  |  |  |  |  |  |
| 9 Phase 7 | -.001 | -.182*** | -.048 | -.040 | -.035 | -.076** | -.060* | -.059* | – |  |  |  |  |  |
| 10 Phase 8 | .012 | -.182*** | -.048 | -.040 | -.035 | -.076** | -.060* | -.059* | -.047 | – |  |  |  |  |
| 11 Phase 9 | -.031 | -.250*** | -.066* | -.055* | -.048 | -.105*** | -.082** | -.081** | -.065* | -.065* | – |  |  |  |
| 12 Phase 10 | -.007 | -.166** | -.044 | -.037 | -.032 | -.070** | -.055* | -.054* | -.043 | -.043 | -.059* | – |  |  |
| 13 Phase 11 | .008 | -.127*** | -.034 | -.028 | -.025 | -.053* | -.042 | -.041 | -.033 | -.033 | -.045 | -.030 | – |  |
| 14 Phase 12^a^ | . | . | . | . | . | . | . | . | . | . | . | . | . | – |

*Note.* Pearson’s correlation coefficient *r*; two-tailed test; Phases defined according to national COVID-19 regulations; Child development was assessed by the ASQ-3 = Ages and Stage Questionnaire-3.

^a^ Could not be calculated because only one father handed in his questionnaire during phase 12 of the COVID-19 pandemic.

**p* < .05; ***p* < .01; ****p* <.001

Supplementary material 2: Intercorrelations between general child development and COVID-19 pandemic-driven adversities of fathers

| **Study variables** | **1** | **2** | **3** | **4** | **5** | **6** | **7** | **8** | **9** | **10** | **11** | **12** | **13** | **14** |
| --- | --- | --- | --- | --- | --- | --- | --- | --- | --- | --- | --- | --- | --- | --- |
| 1 Child development | – |  |  |  |  |  |  |  |  |  |  |  |  |  |
| 2 Before COVID-19 | -.007 | – |  |  |  |  |  |  |  |  |  |  |  |  |
| 3 Phase 1 | -.036 | -.192*** | – |  |  |  |  |  |  |  |  |  |  |  |
| 4 Phase 2 | -.033 | -.158*** | -.041 | – |  |  |  |  |  |  |  |  |  |  |
| 5 Phase 3 | .060 | -.126*** | -.033 | -.027 | – |  |  |  |  |  |  |  |  |  |
| 6 Phase 4 | .056 | -.297*** | -.077* | -.063 | -.050 | – |  |  |  |  |  |  |  |  |
| 7 Phase 5 | .065* | -.224*** | -.058 | -.048 | -.038 | -.089** | – |  |  |  |  |  |  |  |
| 8 Phase 6 | -.075* | -.241*** | -.062 | -.051 | -.041 | -.096** | -.072 | – |  |  |  |  |  |  |
| 9 Phase 7 | .006 | -.181*** | -.047 | -.038 | -.031 | -.072* | -.054 | -.058 | – |  |  |  |  |  |
| 10 Phase 8 | .009 | -.176*** | -.045 | -.037 | -.030 | -.070* | -.053 | -.057 | -.043 | – |  |  |  |  |
| 11 Phase 9 | .005 | -.254*** | -.066* | -.054 | -.043 | -.101** | -.076* | -.082 | -.062 | -.060 | – |  |  |  |
| 12 Phase 10 | -.061 | -.181*** | -.047 | -.038 | -.031 | -.072* | -.054 | -.058 | -.044 | -.043 | -.062 | – |  |  |
| 13 Phase 11 | .013 | -.142*** | -.037 | -.030 | -.024 | -.057 | -.043 | -.046 | -.034 | -.033 | -.048 | -.034 | – |  |
| 14 Phase 12^a^ | . | . | . | . | . | . | . | . | . | . | . | . | . | – |

*Note.* Pearson’s correlation coefficient *r*; two-tailed test; Phases defined according to national COVID-19 regulations; Child development was assessed by the ASQ-3 = Ages and Stage Questionnaire-3.

^a^ Could not be calculated, because only one father handed in his questionnaire during phase 12 of the COVID-19 pandemic, therefore the variable was constant.

**p* < .05; ***p* < .01; ****p* <.001

Supplementary material 3: Mediation Analyses (with outliers)

|  | **(c) Total effect of X on Y** | | | | | **(c’) Direct effect of X on Y** | | | | | **(ab) Indirect effect of X on Y** | | | | |
| --- | --- | --- | --- | --- | --- | --- | --- | --- | --- | --- | --- | --- | --- | --- | --- |
|  | ***b*** | ***SE*** | ***β*** | ***p*** | ***BCa 95% CI*** | ***b*** | ***SE*** | ***β*** | ***P*** | ***BCa 95% CI*** | ***b*** | ***SE*** | ***β*** | ***BCa 95% CI*** |  |
|  | **Mothers** | | | | | | | | | | | | | | |
| 1^a^ | -0.606 | 0.422 | -.039 | .151 | [-1.435; 0.222] | -0.387 | 0.426 | -.025 | .363 | [-1.223; 0.447] | - 0.219* | 0.093 | -.014* | [-0.417; -0.049] |  |
| 2^b^ | -0.367 | 0.428 | -.024 | .391 | [-1.206; 0.472 | -0.172 | 0.425 | -.011 | .686 | [-1.001; 0.662] | -0.195* | 0.083 | -.013* | [-0.376; -0.049] |  |
| 3^c^ | -0.430 | 0.449 | -.028 | .338 | [-1.311; 0.451] | -0.397 | 0.446 | -.026 | .374 | [- 1.273; 0.479] | -0.033 | 0.048 | -.002 | [-0.137; 0.053] |  |
|  | **Fathers** | | | | | | | | | | | | | | |
| 1^a^ | -0.574 | 0.737 | -.027 | .437 | [-2.021; 0.874] | -0.040 | 0.739 | -.002 | .956 | [-1.490; 1.409] | -0.533* | 0.179 | -.025* | [-0.951; -0.236] |  |
| 2^b^ | 0.545 | 0.846 | .025 | .519 | [-1.116; 2.207] | -0.938 | 0.839 | .043 | .264 | [-0.708; 2.585] | -0.393* | 0.176 | -.019* | [-0.789; -0.104 |  |
| 3^c^ | 1.096 | 0.871 | .049 | .209 | [-.613; 2.806] | 1.186 | 0.857 | .054 | .167 | [-.497; 2.869] | -0.089 | 0.114 | -.004 | [-0.354; 0.098] |  |

*Note.* Mediation analysis conducted with prenatal anxiety symptoms as predictor (X), parent-infant-bonding as mediator (M) and early child development as outcome (Y). Confounders included were: pregnancy complications, breastfeeding, child sex, preterm birth, social support, partnership satisfaction, education, and COVID-19 phase 5 and 6. In the third analysis postnatal depression symptoms were included additionally. BCa 95% CI = Bias corrected and accelerated 95 % bootstrap confidence interval.

^a^ Model 1 = Only main variables

^b^ Model 2 = With confounders, but without postnatal depression symptoms

^c^ Model 3 = With confounders and postnatal depression symptoms

**Supplementary material 4*:*** *Mediation analyses:* Pathways a and b (with outliers)

|  | **(a) Effect of X on M** | | | | | **(b) Effect of M on Y** | | | | |
| --- | --- | --- | --- | --- | --- | --- | --- | --- | --- | --- |
|  | ***b*** | ***SE*** | ***β*** | ***p*** | ***BCa 95% CI*** | ***b*** | ***SE*** | ***β*** | ***p*** | ***BCa 95% CI*** |
|  | **Mothers** | | | | | | | | | |
| 1^a^ | 0.611 | 0.091 | .188 | < .001 | [0.432; 0.7899] | -0.358 | 0.138 | -.075 | .009 | [-0.628; -0.088] |
| 2^b^ | 0.504 | 0.095 | .154 | < .001 | [0.316; 0.691] | -0.387 | 0.143 | -.083 | .007 | [-0.667; -0.106] |
| 3^c^ | 0.069 | 0.093 | .021 | .462 | [-0.114; 0.251] | -0.482 | 0.159 | -.104 | .002 | [-0.793; -0.171] |
|  | **Fathers** | | | | | | | | | |
| 1^a^ | 0.673 | 0.146 | .172 | < .001 | [0.387; 0.959] | -0.792 | 0.182 | -.146 | < .001 | [-1.149; -0.434] |
| 2^b^ | 0.619 | 0.169 | .152 | < .001 | [0.287; 0.950] | -0.635 | 0.206 | -.117 | .002 | [-1.039; -0.232] |
| 3^c^ | 0.159 | 0.178 | .039 | .367 | [-.188; 0.507] | -0.56 | 0.222 | -.104 | .012 | [-0.995; -0.126] |

*Note.* Mediation analysis conducted with prenatal anxiety symptoms as predictor (X), parent-infant-bonding as mediator (M) and early child development as outcome (Y). Confounders included were: pregnancy complications, breastfeeding, child sex, preterm birth, social support, partnership satisfaction, education, and COVID-19 phase 5 and 6. In the third analysis postnatal depression symptoms were included additionally. BCa 95% CI = Bias corrected and accelerated 95 % bootstrap confidence interval.

^a^ Model 1 = Only main variables

^b^ Model 2 = With confounders, but without postnatal depression symptoms

^c^ Model 3 = With confounders and postnatal depression symptoms

**Supplementary material 5:** Mediation Analyses without outliers

|  | **(c) Total effect of X on Y** | | | | | **(c’) Direct effect of X on Y** | | | | | **(ab) Indirect effect of X on Y** | | | | |
| --- | --- | --- | --- | --- | --- | --- | --- | --- | --- | --- | --- | --- | --- | --- | --- |
|  | ***b*** | ***SE*** | ***β*** | ***p*** | ***BCa 95% CI*** | ***b*** | ***SE*** | ***β*** | ***p*** | ***BCa 95% CI*** | ***b*** | ***SE*** | ***β*** | ***BCa 95% CI*** |  |
|  | **Mothers** | | | | | | | | | | | | | | |
| 1^a^ | -0.799 | .450 | -.049 | .076 | [-1.683; 0.084] | -0.594 | .460 | -.037 | .197 | [-1.497; 0.308 | -0.205 | .093 | -.013 | [-0.398; -034] |  |
| 2^b^ | 0.284 | .415 | -.019 | .493 | [-1.098; 0.529] | -0.127 | .413 | -.008 | .758 | [0.683; -0.008] | -0.157 | .076 | -.010 | [-0.326; -0.028] |  |
| 3^c^ | -0.367 | .435 | -.024 | .339 | [-1.220; 0.487] | -0.348 | .433 | -.023 | .421 | [-1.197; 0.501] | -0.019 | .042 | -.001 | [-0.111; 0.056] |  |
|  | **Fathers** | | | | | | | | | | | | | | |
| 1^a^ | -0.829 | .783 | -.036 | .289 | [-2.366; 0.707] | -0.415 | .782 | -.018 | .596 | [-1.949; 1.119] | -0.414 | .150 | -.018 | [-0.749; -0.166] |  |
| 2^b^ | 0.338 | .877 | .015 | .698 | [-1.370; 2.046] | 0.645 | .877 | -029 | .462 | [-1.075; 0.366] | -0.307 | .152 | -.014 | [-0.667; -0.074] |  |
| 3^c^ | 1.071 | .887 | .047 | .228 | [-0.671; 2.812] | 1.083 | .879 | .048 | .218 | [-0.642; 2.807] | -0.012 | .085 | -.001 | [-0.200; 0.154] |  |

*Note.* Mediation analysis conducted with prenatal anxiety symptoms as predictor (X), parent-infant bonding as mediator (M) and early child development as outcome (Y). Confounders included were: pregnancy complications, breastfeeding, child sex, preterm birth, social support, partnership satisfaction, education, Covid-19 phase 5 and 6; In the third analysis postnatal depression symptoms were included additionally. BCa 95% CI = Bias corrected and accelerated 95 % bootstrap confidence interval.

^a^ Model 1 = Only main variables

^b^ Model 2 = With confounders, but without postnatal depression symptoms

^c^ Model 3 = With confounders and postnatal depression symptoms

**Supplementary material 6:** Mediation analyses: Pathways a and b without outliers

|  | **(a) Effect of X on M** | | | | | **(b) Effect of M on Y** | | | | |
| --- | --- | --- | --- | --- | --- | --- | --- | --- | --- | --- |
|  | ***b*** | ***SE*** | ***β*** | ***p*** | ***BCa 95% CI*** | ***b*** | ***SE*** | ***β*** | ***p*** | ***BCa 95% CI*** |
|  | **Mothers** | | | | | | | | | |
| 1^a^ | 0.645 | .090 | .195 | < .001 | [0.468; 0.822] | -0.318 | .137 | -.065 | .021 | [-.587; -.048] |
| 2^b^ | 0.494 | .095 | .148 | <. 001 | [0.308; 0.679] | -0.318 | .138 | -.070 | .021 | [-0.589; -0.047] |
| 3^c^ | 0.043 | .090 | .013 | .635 | [-0.134; 0.220] | -0.437 | .157 | -0.095 | .005 | [-0.744; -0.129] |
|  | **Fathers** | | | | | | | | | |
| 1^a^ | 0.623 | .127 | .151 | < .001 | [0.374; 0.876] | -0.665 | .187 | -.118 | < .001 | [-1.032; -0.298] |
| 2^b^ | 0.485 | .152 | .117 | .002 | [0.186; 0.783] | -0.634 | .221 | -.119 | .004 | [-1.068; -0.200] |
| 3^c^ | 0.024 | .157 | .006 | .878 | [-0.284; 0.332] | -0.499 | .219 | -.093 | .023 | [-0.929; -0.069] |

*Note.* Mediation analysis conducted with prenatal anxiety symptoms as predictor (X), parent-infant bonding as mediator (M) and early child development as outcome (Y). Confounders included were: pregnancy complications, breastfeeding, child sex, preterm birth, social support, partnership satisfaction, education, Covid-19 phase 5 and 6; In the third analysis postnatal depression symptoms were included additionally. BCa 95% CI = Bias corrected and accelerated 95 % bootstrap confidence interval

^a^ Model 1 = Only main variables

^b^ Model 2 = With confounders, but without postnatal depression symptoms

^c^ Model 3 = With confounders and postnatal depression symptoms

Supplementary material 7: Standardized regression coefficients and p-values of all confounders in pathway a

| **Outcome: Parent-child bonding^a^** | | **Mothers** | | **Fathers** | |
| --- | --- | --- | --- | --- | --- |
| **Model** | **Predictor** | ***β*** | ***p*** | ***β*** | ***p*** |
| **1** | Prenatal anxiety symptoms^b^ (T1) | .188 | < .001 | .172 | < .001 |
| **2** | Prenatal anxiety symptoms^b^ (T1) | .154 | < .001 | .152 | < .001 |
|  | Pregnancy complications (T2) | -.019 | < .001 | -.017 | .594 |
|  | Breastfeeding (T2) | .029 | .4707 | .006 | .843 |
|  | Child sex (T2) | -.034 | .209 | .013 | .719 |
|  | Preterm birth (T2) | -.006 | .202 | -.018 | .433 |
|  | Social support^c^ (T1) | -.121 | < .001 | -.108 | .004 |
|  | Partnership satisfaction^d^ (T2) | -.095 | < .001 | -.119 | .002 |
|  | Education (T1) | .162 | < .001 | .128 | < .001 |
|  | COVID-19 pandemic phase 5 | -.065 | .0026 | -.054 | .058 |
|  | COVID-19 pandemic phase 6 | -.0266 | .278 | -.037 | .224 |
| **3** | Prenatal anxiety symptoms^b^ (T1) | .021 | .462 | .039 | .367 |
|  | Pregnancy complications (T2) | -.058 | .018 | -.036 | .247 |
|  | Breastfeeding (T2) | .029 | .162 | .012 | .669 |
|  | Child sex (T2) | -.033 | .167 | .006 | .852 |
|  | Preterm birth (T2) | .008 | .742 | -.011 | .636 |
|  | Social support^c^ (T1) | -.064 | .019 | -.067 | .072 |
|  | Partnership satisfaction^d^ (T2) | -.053 | .028 | -.063 | .091 |
|  | Education (T1) | .175 | < .001 | .131 | < .001 |
|  | COVID-19 pandemic phase 5 | -.051 | .004 | -.047 | .077 |
|  | COVID-19 pandemic phase 6 | -.024 | .285 | -.050 | .075 |
|  | Postnatal depression symptoms^e^ (T2) | .421 | < .001 | .359 | < .001 |

*Note.* T1, during late pregnancy; T2, eight weeks postpartum; T3, 14 months postpartum.

^a^PBQ, Postpartum Bonding Questionnaire; ^b^SCL-90-R, Symptom Check List 90 Revised; ^c^F-SozU-14, Short version of the Social Support questionnaire (German: Fragebogen zur Sozialen Unterstützung); ^d^PFB-K, Short Version of the Partnership questionnaire (German: Kurzform des Partnerschaftsfragebogens); ^e^EPDS, Edinburgh Postnatal Depression Scale.
